# Imre Festetics and the Sheep Breeders' Society of Moravia: Mendel's Forgotten “Research Network”

**DOI:** 10.1371/journal.pbio.1001772

**Published:** 2014-01-21

**Authors:** Péter Poczai, Neil Bell, Jaakko Hyvönen

**Affiliations:** 1 Department of Biosciences, University of Helsinki, Helsinki, Finland; 2 Botanical Museum, University of Helsinki, Helsinki, Finland

## Abstract

Poczai and coauthors introduce us to a Moravian Count, Imre Festetics, who formulated many principles of genetics before Mendel was born and helped establish the “research network” within which Mendel later developed his own ideas.

## Summary

Contemporary science thrives on collaborative networks, but these can also be found elsewhere in the history of science in unexpected places. When Mendel turned his attention to inheritance in peas he was not an isolated monk, but rather the latest in a line of Moravian researchers and agriculturalists who had been thinking about inheritance for half a century. Many of the principles of inheritance had already been sketched out by Imre Festetics, a Hungarian sheep breeder active in Brno. Festetics, however, was ultimately hindered by the complex nature of his study traits, aspects of wool quality that we now know to be polygenic. Whether or not Mendel was aware of Festetics's ideas, both men were products of the same vibrant milieu in 19th-century Moravia that combined theory and agricultural practice to eventually uncover the rules of inheritance.

## Introduction

“*For your own, work with tireless efforts if you want to understand what are the rules imposed by nature to itself.*”

Imre Festetics

**Figure 1:**
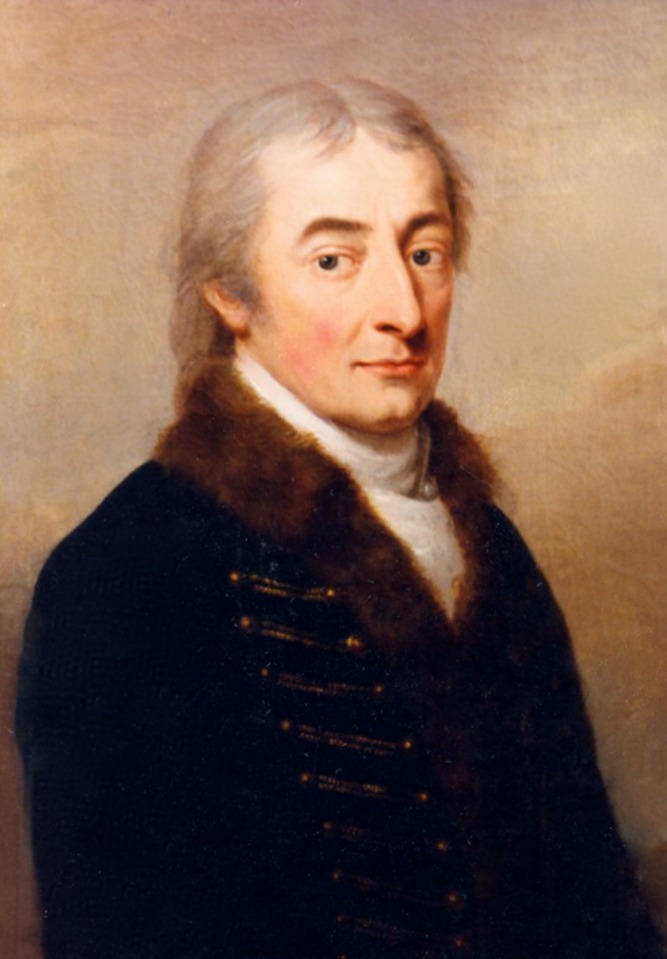
Count Imre Festetics around 1819. Portrait by Oelenhainz August Friedrich, original painting found in Kőszeg City Museum (No. 55.11).

Most students are still taught that the discipline of genetics began with Mendel, and would be surprised to learn that many of the central principles were formulated before Mendel was born, also in Brno where Mendel later worked, and through the study of sheep rather than peas. Inasmuch as a single individual can be credited for pre-Mendelian genetics, it is Count Imre (Emmerich) Festetics [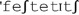] (1764–1847), a sheep breeder based in Hungary, who remains as obscure today as Mendel is famous. Festetics himself (Figure 1) was very much the product of a well-established intellectual environment that had arisen in Moravia (now part of the Czech Republic) in the late 18th century—a vigorous crucible of practically minded but often highly educated agriculturalists with collective access to a wide range of material and financial resources.

Livestock farmers had always had an interest in breeding and an awareness of the importance of parentage or “blood.” Before the mid-18th century, however, it was generally believed that climate, soil, etc.—factors we would today call “environmental”—had by far the largest influence on the characteristics of animals in a given region over multiple generations. While it was known that breeds could be improved by crossing with animals from elsewhere with desirable traits, the gradual deterioration of the introduced features in subsequent generations was seen as evidence for the dominant influence of local conditions, or “pasture.” To some extent this inhibited experimentation with breeding, as the advantages gained from crossing were seen as inherently temporary.

**Figure 2:**
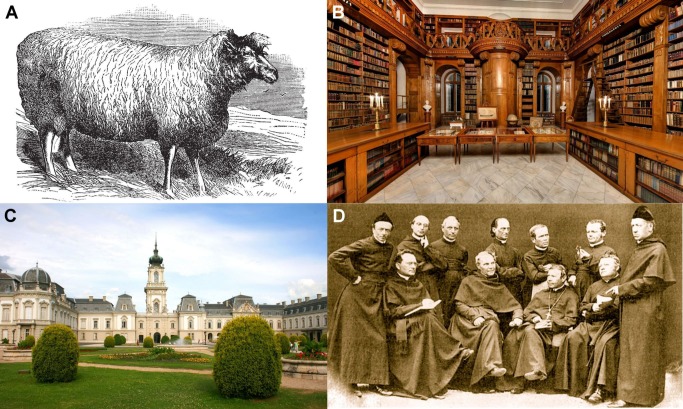
Historic photos. (A) Robert Bakewell's barrel-shaped New Leicester (Dishley) ram, created through inbreeding on his farm at Dishley, Leicestershire. Vintage engraved illustration, Trousset Encyclopaedia (1886–1891). (B) The enormous Festetics family library, consisting of 90,000 volumes and the only aristocratic library remaining in Hungary. It nowadays operates as a museum, open to visitors. Photo kindly provided by 3dpano.hu. (C) The Festetics castle at Keszthely on Lake Balaton where the family library is housed. Imre Festetics read and studied here, despite living and sheep-breeding in the city of Kőszeg. Photo kindly provided by 123rf.com. (D) Members of St. Thomas's Abbey in Brno about 1862. Gregor Mendel is standing second from right, while Cyrill Napp is seated second from right. Photo kindly provided by Jiří Sekerák from the archive of Mendelianum, Moravian Museum, Brno, Czech Republic.

This point of view began to change in the late 18th century as some breeders experienced dramatically increased success in producing animals ideally suited to commercial purposes, such as meat or wool production, and were able to maintain such breeds indefinitely without apparent degeneration. By far the most successful of these was the English sheep breeder Robert Bakewell (1725–1795), whose famous “New Leicester” sheep (Figure 2A) had a barrel-like form effectively designed to maximize the quantity of meat obtained for a given amount of feeding. Bakewell's success lay in his highly methodical approach to close inbreeding (inbreeding with first-degree relatives), and helped to encourage the developing belief that breed was more important than “pasture.” With the correct approach, it now seemed, the “blood” of certain animals could become “fixed” for certain desirable traits [1].

Bakewell gained a formidable reputation both nationally and internationally for having effectively harnessed the power of “heredity” [1], although the concept itself had yet to be formulated in a manner that we would recognize today [2]. He was, however, highly secretive about his methods, and although well-read in practical matters he was no academic and certainly had no ambitions to investigate abstract laws of nature. Uncovering the laws of inheritance would itself require a successful cross-fertilisation between the accumulated practical experience of agriculturalists and the scientific establishment. Such a meeting of minds was unlikely in England, where the eminent naturalist Sir Joseph Banks regarded Bakewell with suspicion [2], and it would be the application of Bakewell's methods to wool production in Moravia by Ferdinand Geisslern (1751–1824) that would eventually bring them to the attention of a scientifically minded audience.

## The Sheep Breeders' Society in Moravia

Brno, the capital city of Moravia, (Brünn, in Mähren, to Germans) had developed as the center of a thriving wool industry in the 18th century and was to become known as “the Austrian Manchester,” in reference to the age-old preeminence of Manchester in English wool production. Local sheep breeders in Brno founded the world's first animal breeding society, known rather grandly as “The Association of Friends, Experts and Supporters of Sheep Breeding for the achievement of a more rapid and more thoroughgoing advancement of this branch of the economy and the manufacturing and commercial aspects of the wool industry that is based upon it,” but usually called the Sheep Breeders' Society (SBS) (*Schafzüchtervereinigung*). The SBS focused on practical problems in the wool industry and published a weekly journal, *Oekonomische Neuigkeiten und Verhandlungen* (ONV; Economic News and Announcements), edited by the society secretary Christian Carl André (1763–1832), the leading figure in the development of natural and agricultural sciences in Moravia at that time. The annual meetings attracted a wide range of participants, not only from Moravia but also from the neighboring regions of Hungary, Bohemia, and Silesia. Many members had extensive libraries of scientific books and journals; Count H.F. Salm-Reifferscheidt's (1778–1836) collection, for example, numbered 59,000 volumes. André and his family had full access to these resources, and his son Rudolf (1792–1825) was later to write a book on sheep breeding.

Imre Festetics was another notable member of the society and had access to a huge library (Figure 2B) of agricultural books owned by his elder brother (György Festetics, 1755–1819) housed in their castle (Figure 2C) at Keszthely on Lake Balaton. It included the works of Young, Culley, Sinclair, and Marshall, as well as the county surveys of the Board of Agriculture in London [3],[4], the same publications that had influenced Bakewell. On the basis of this knowledge, György Festetics founded the Georgikon University (1797), the first agricultural college in Europe, and still extant as a faculty of the University of Pannonia. The SBS undoubtedly brought together an unusually progressive-thinking group of people interested in the advancement of the textile industry through the improvement of wool traits in sheep. The annual meetings were true scientific melting pots of their time, and a long-forgotten focus of great scientific debate and discovery.

## Sheep Inbreeding: The Big Debate

Between 1816 and 1819, members of the SBS extensively debated the association of wool traits (color, fitness, density, etc.), and how to effectively combine useful traits in the progeny of crosses [1]. The most controversial topic was the role of inbreeding. The Austrian Baron J. M. Ehrenfels maintained that “heredity” was controlled by “physiological laws of nature” (*physiologische Gesetze der Natur*), illustrating his point with reference to the Spanish Merino breed. The quality of the wool had been observed to decrease when sheep were bred outside of Spain, and Ehrenfels attributed this to climatic conditions. He also believed that inbreeding would act against the “main plasma” of animal organization (*Hauptplasma der thierischen Organisation*) directly decreasing wool fitness [5]. Contrary to Ehrenfels, Imre Festetics believed that heredity was strictly controlled by intrinsic factors, and inbreeding could be used to concentrate these factors and make the inheritance of traits more predictable. His hypotheses were based on his own practical experiences and his observations of Merino sheep breeding. Influenced by Bakewell's approach—often referred to as “breeding in-and-in”—which had rapidly gained popularity with European breeders interested in fine wool production, Festetics had started to breed sheep in 1803 on his estate in Hungary. After experimenting with rigorous inbreeding methods for more than a decade he had reached the point where he was unable to buy better stock animals than his own. Others turned their attention to his results, which he publicized at meetings of the SBS, often humorously referring to them as *Brünni Juhos Társaság*, or “the Brno sheepy bunch.” [6].

André attempted to resolve the debate between Ehrenfels and Festetics. He agreed on the value of inbreeding as proposed by Festetics, although he had doubts about its potential, and asked Festetics to summarize his points in a paper similar to that of Ehrenfels. Festetics accepted the challenge, confident that his 15 years of breeding experience would enable him to back up his claims. This resulted in a series of papers published in 1819 [7],[8].

## Genetic Laws of Nature

Festetics formulated a number of rules of heredity and was the first to refer to these as “genetic laws of nature” (“*Die genetische Gesätze der Natur*”). In so doing he used the term “genetic” for the first time, 80 years before William Bateson did so in his personal letter to Alan Sedgwick. Festetics created this new term to clearly distinguish his rules of heredity, or “genetic laws,” from the “physiological laws” of Ehrenfels. Festetics's four rules (originally in German) may be translated as follows:

- Healthy and robust animals are able to propagate and pass on their specific characteristics.
- Traits of grandparents that are different from those of the immediate progeny may reappear in later generations.
- Animals possessing desirable traits that have been inherited over many generations can sometimes have offspring with divergent traits. Such progeny are variants or freaks of nature, and are unsuitable for further propagation if the aim is the heredity of specific traits.
- A precondition for successful application of inbreeding is scrupulous selection of stock animals. (In my opinion this is the main point). [Footnote inserted by C.C. André].

In these “Genetic Laws,” Festetics was the first to recognize empirically the segregation of characters in the second hybrid generation [9]. He also linked heredity (*Vererbung*) with health and vigor independently of external factors, stressing the role of inbreeding (combined with strong selection) in stabilizing character inheritance for preserving or developing new races [10]. To illustrate the concept he used sheep and horse breeds as examples, although he also applied it to the human species by considering populations of isolated Hungarian villages, in which he had observed degenerative mental and physical characteristics. Festetics's observations highlighted important correlations between variability, adaptation, and development. He also noted the consequences of selection and its role in heredity, believing that variability and his postulated laws of genetics were connected, acting together in breeding as well as in the natural processes controlling populations of different animals, including humans.

## The Impact of Festetics

There is no doubt that Festetics's laws were derived empirically and arose mainly from the practical need to produce sheep with better wool traits. Although there was initially no attempt to represent them mathematically, it appears from his later publications that Festetics was aware of the importance of applying such methods. Rudolf André designed a micrometer device that could be used to evaluate different wool traits. Festetics reacted by stating, “I believe that in breeding science a new era is about to emerge, starting with the fine measurement of wool traits that can be evaluated with mathematical accuracy.” In a paper published a year later he argued for the importance of applying mathematical evaluations in animal breeding [11].

At this time “heredity” as such had no biological meaning [2]. As Sandler and Sandler [12] point out, there was as yet no clear distinction between the concepts of heredity and development. Contemporary scientists regarded heredity as a stage in a seamless process of development, and never considered that events of transmission could be detached and studied separately [13]. Festetics's laws show that he was very close to making this crucial distinction between inheritance *sensu stricto* and “development” *sensu lato*. Unfortunately, his traits of choice, such as wool density and length, were complex and subject to polygenic inheritance. To fully quantify his observations and reach the same conclusions as Mendel later did with monogenic traits, it would have been necessary for Festetics to have had access to precise techniques and modern statistical methods such as quantitative trait loci (QTL) mapping [14]. Thus any attempt to analyse his data using 19th-century methods would have led him to a dead end.

The inevitable question arises as to whether Festetics and Mendel were aware of each other's work. There is no direct evidence that Mendel ever read or cited the work of Festetics, despite it being available in the library in Brno where he did his research. However, Mendel's law of segregation is essentially the mathematical proof of Festetics' rule “b.” Mendel's law states that during the production of gametes, the two copies of each hereditary factor segregate such that the offspring acquire only one factor from each parent. Mendel proved this by the observation of reappearance of the grandparents' traits in the second generation of peas. Is this coincidental, or did Mendel precisely design an experiment to prove a previous empirical observation? Although the two men were a generation apart, some of the answers to the questions Mendel was asking were in the library that he used continuously. Both Festetics and Mendel were members of the Natural History Society in Brno at the same time, though their overlap was brief, as Festetics died only a year after Mendel became a member. In 1865 Mendel read his paper at the annual meeting of the society and it was published a year later in the proceedings [15]. Although we will never know whether Festetics directly influenced Mendel, both men were products of the same community, and Mendel may well have been aware of Festetics' ideas. The *modus operandi* of this community closely mirrored that of modern collaborative research networks, with powerful and well-funded individuals able to bring together scientists from different disciplines to answer specific questions with profound theoretical and commercial implications.

## “What is inherited and how?”

In 1820 C.C. André, the leading figure in shaping the intellectual environment of the SBS, moved to Stuttgart, leaving Johann Karl Nestler (1783–1842) to refocus the debate a few years later on attempts to understand inheritance. Nestler conducted extensive animal and plant heredity experiments and was head of the Department of Natural History and Agriculture at the University of Olomouc, where Mendel had previously studied. In 1836, more than a decade after the debate on Festetics's “genetic laws of nature,” an impromptu meeting of the SBS was held to discuss “the inheritance capacity of noble stock animals.” One speaker was the new abbot of St. Thomas' Abbey, Cyrill Franz Napp (1792–1867). Napp made the important observation that “…heredity of characteristics from the producer (*Erzeuger*) to the produced (*Erzeugten*) consists above all in the mutual affinity by kinship of paired animals. As a result of this, a ram chosen for the ewe should correspond to it in both inner and outer organization. [16].” He thus recognized a role for the “inner organization” of animals in determining their “outer forms,” and later went on to ask a critical question: “What we should have been dealing with is not the theory and process of breeding. But the question should be: *what is inherited and how* [17]?” He was effectively formulating the topic of the plant genetic research later to be carried out by Mendel. Inspired by this, Nestler called for crossing experiments specifically designed to address these ideas [18],[19], and included the word “heredity” in the title of a book he published in 1837 [20].

Many researchers have attempted to reveal the motivation for Mendel's experimental design [21],[22]. Was he interested in the theoretical underpinning of the laws of heredity or simply aiming to create hybrids? Monaghan and Corcos [23] question the influence of Moravian breeders on Mendel's experiments. However, as shown by Wood and Orel [19], it is clear that many intellectuals in Brno were actively debating both theoretical and empirical issues related to heredity. The Abbey of St. Thomas was well equipped for scientific research, and most of the friars enjoyed a rich intellectual life [22]. Napp was Mendel's mentor in Brno (Figure 2D), actively promoting the teaching of agriculture and giving lectures that Mendel attended in 1846 [21]. Mendel's teachers had a strong influence on him, with Napp effectively headhunting him for the monastery in 1843 [19],[24]. Napp was interested in heredity as a problem in itself, and sent Mendel to the University of Vienna to gain specific expertise in 1851–1853. Then he set him to work on the question of the nature of heredity. Napp and Nestler were principle figures shaping heredity research in Brno. Both had read Festetics's papers and cited him, and it seems probable that Mendel would have heard about these works from his teachers.

## Conclusions

Although teetering on the brink of insight, Festetics's work did not immediately lead to a great breakthrough in our understanding of heredity [2]. Instead it sunk into complete obscurity for more than 170 years until its rediscovery by Orel [25]. Festetics did not discover factorial or “Mendelian” genetics before Mendel, but he certainly laid the groundwork for their later discovery [9]. If Mendel is the father of genetics in this context, Festetics has a strong claim to be the grandfather, having introduced the term “genetic” as early as 1819. Unfortunately, with a few exceptions, Festetics is rarely mentioned in contemporary scientific literature or in books addressing the history of genetics. Nonetheless, he made an enormous contribution to the intellectual context in Brno from which Mendel's interest in heredity and hybridization arose. Mendel—while undoubtedly highly talented—was not a “lone genius” any more than Festetics was. Both men were part of a scientific community—effectively a research network—engaged in solving the problem of heredity. The work begun by Festetics appeared to have reached an impasse until Mendel arrived in Brno and, whether by accident or design, selected the right tool for the job—peas with discrete characters and shorter generation times than sheep.
